# Efficacy of Lactose Reduction in Irritable Bowel Syndrome and Recurrent Abdominal Pain: A Systematic Review

**DOI:** 10.3390/nu18213619

**Published:** 2026-10-31

**Authors:** Sébastien Kindt, Marieke Robbrecht, Lisa Van den Eeckhaut, Mieke Van der Merwe, Hanne Delcourt, Chloé Melchior, Guillaume Gourcerol, Yvan Vandenplas, Koen Huysentruyt

**Affiliations:** 1 Department of Gastroenterology and Hepatology, UZ Brussel, Vrije Universiteit Brussel (VUB), B-1090 Brussels, Belgium; lvdeeckhaut@outlook.com (L.V.d.E.); mieke.vandermerwe@uzbrussel.be (M.V.d.M.); 2 Kindergastro-Enterologie, Hepatologie en Nutritie, KidZ Health Castle, UZ Brussel, Vrije Universiteit Brussel (VUB), B-1090 Brussels, Belgium; mariekerobbrecht@gmail.com (M.R.); hanne.delcourt@uzbrussel.be (H.D.); yvan.vandenplas@uzbrussel.be (Y.V.); koen.huysentruyt@uzbrussel.be (K.H.); 3 Univ Rouen Normandie, Normandie Univ, ADEN UMR1073, Nutrition, Inflammation and Microbiota-Gut-Brain Axis, CHU Rouen, Department of Gastroenterology, INSERM CIC-CRB 1404, F-76000 Rouen, France; chloe.melchior@chu-rouen.fr; 4 Univ Rouen Normandie, Normandie Univ, ADEN UMR1073, Nutrition, Inflammation and Microbiota-Gut-Brain Axis, CHU Rouen, Department of Physiology, INSERM CIC-CRB 1404, F-76000 Rouen, France; guillaume.gourcerol@chu-rouen.fr; 5 Mental Health and Wellbeing Research Group, Vrije Universiteit Brussel (VUB), B-1090 Brussels, Belgium

**Keywords:** irritable bowel syndrome, recurrent abdominal pain, lactose, breath test, systematic review

## Abstract

Background/Objectives: Lactose intolerance (LI), irritable bowel syndrome in adults (IBS) and recurrent abdominal pain (RAP) in children are frequently diagnosed worldwide. This systematic review aimed to assess the effects of dietary reduction in lactose on symptoms, quality of life and healthcare visits in IBS and children with recurrent abdominal pain. Methods: PubMed, Cochrane Library, Embase and Lilacs were searched for studies on IBS and RAP investigating the effect of a reduction of dietary intake of lactose for at least 4 weeks on abdominal pain, stool characteristics, bloating and distension, and quality of life published up to 15 August 2026. No restriction in language, publication type or data limit was applied. Four reviewers independently screened the abstracts for eligibility, and risk of bias was assessed by two reviewers with ROBINS-2. Disagreements were resolved by consensus or consulting with a senior investigator. Results were summarised in tabular form. The systematic review was registered on PROSPERO (CRD42024617161). Results: Eighteen studies met eligibility criteria based on the title and abstract. Upon review of full-text articles, three studies in adults and six studies in children were retained for data extraction. Lactose breath tests (LBTs) were performed in all studies. All but one study prospectively compared patient cohorts. IBS was defined by Manning criteria in two studies and Rome II criteria in one study. Only patients with a positive LBT were subjected to dietary intervention in eight of nine studies. No control group was included in one of three and three of six of the studies in adults and children, respectively. None of the studies compared symptomatic evolution based on validated questionnaires. Improvement in pain was reported by two of three and five of six of the studies on IBS and RAP, respectively. The risk of bias was high for all studies. No study assessed quality of life. Conclusions: Current evidence in support of dietary lactose reduction in patients with IBS and RAP is limited due to a high risk of bias, the use of old criteria, the absence of a valid comparator group and poorly defined outcomes. A positive LBT was associated with improvement in pain after a lactose-free diet. Future studies on the role of lactose restriction in IBS and RAP should recruit patients based on current diagnostic criteria and assess outcome by validated measures, preferentially applying a randomised controlled design.

## 1. Introduction

Lactose intolerance (LI) and irritable bowel syndrome (IBS) are frequently diagnosed worldwide. LI is an organic disorder. It is diagnosed when lactose malabsorption (LM) causes symptoms. IBS, on the other hand, is part of the disorders of gut–brain interactions. Its diagnosis relies on symptom-based criteria in the absence of a readily identifiable organic cause [1]. Prior to the recent harmonisation between paediatric and adult populations [2], abdominal pain without apparent cause in children was referred to as recurrent abdominal pain (RAP). With the inclusion of the paediatric population and harmonisation with the terminology in adults, RAP has been deprecated and is now largely considered IBS, as defined by the Rome V criteria [2].

The prevalence of LM remains uncertain. A publication from 2017, that has been retracted in 2025, estimated the global prevalence of LM at 68%, but this was subject to large regional variations, with the highest numbers in the Middle East. Depending on the defining criteria, the prevalence of IBS is estimated at 4–10% in the general population worldwide [3]. In children, recurrent abdominal pain (RAP) has an estimated pooled prevalence of 11.7%, although it was observed in at least 26.2% of the Swedish paediatric population at one time point [4,5]. Clinically, abdominal symptoms, such as pain and bloating, and/or alterations in stool form or frequency are all symptoms that may be present in LM, IBS and RAP.

LM is diagnosed based on the results of a lactose breath test either based on respiratory excretion of hydrogen or ^13^C- or ^14^C-marked carbon dioxide. The North American and European consensus and the European Society for Paediatric Gastroenterology, Hepatology, and Nutrition (ESPGHAN) provided a basis for the standardisation of the hydrogen breath test (H_2_BT) across different centres [6,7,8]. All recommend testing with a dose of 25 g of lactose in adults and 0.5–2 g/kg to a maximum of 25 g up to 50 g in children. A rise in H_2_ and CH_4_ from baseline determines the presence of malabsorption. Apart from the breath test results, the European guidelines advice is to simultaneously record symptoms experienced by patients during the test, e.g., with the paediatric or adult carbohydrate perception questionnaire [9,10]. If LM is accompanied by symptoms, it is termed LI. In contrast, in the absence of a biomarker, the diagnosis of IBS relies on symptom-based criteria, such as the Rome IV and the more recent Rome V criteria, in which the criteria for IBS have become more aligned between children and adults [1,11].

Current evidence does not support LI as a cause of IBS or RAP. The prevalence of LM is similar in IBS and the general population [12]. Studies in adults indicated that the prevalence of LM and LI remains unaffected by the presence of IBS [13,14]. Nevertheless, IBS patients frequently report food intolerances [15]. In a prospective study of 62 Indian children meeting the criteria for RAP, 58% suffered from LI [16]. According to a meta-analysis, IBS patients more frequently report symptoms after ingestion of lactose irrespective of LM, possibly indicative of a lower lactose threshold for symptom generation [12,17]. This is in line with the beneficial effects of a so-called low FODMAP (fermentable oligo-, di-, monosaccharides and polyols) diet. FODMAPs can elicit symptoms in susceptible individuals as a result of fermentation and osmotic activity in the gut. The low-FODMAP diet restricts the intake of fructose, fructans, galacto-oligosaccharides and polyols (sugar alcohols), as well as the disaccharide lactose. Different studies confirmed its efficacy in IBS in terms of abdominal pain, stool consistency and bloating [18,19], with FODMAP-like diets even being superior to treatment with spasmolytics [20]. It is conceivable that lactose restriction, as a FODMAP, will result in improvement of IBS in some patients. However, a small study in children with RAP did not report improvement of pain symptoms on a lactose-free diet [21]. In adults with IBS, a systematic review of six studies pointed to the high rate of false positives when assessing self-reported symptoms during an LBT. It proved challenging to differentiate symptoms between IBS and LI. The review also concluded that LBT should not be routinely recommended [22]. Similar conclusions were drawn in a narrative review on dietary interventions for IBS [19]. However, in both reviews the symptomatic response to LFD remained unaddressed. Therefore, it remains uncertain whether an LFD should be recommended to address specific symptoms of IBS or RAP.

This systematic review aimed to objectify the effectiveness of lactose restriction in reducing symptoms of IBS and RAP. To address this goal, the primary endpoint consisted of an assessment of the existing evidence of dietary reduction of lactose on abdominal pain. Secondary endpoints evaluated the effect on other symptoms, quality of life and healthcare visits in adults with IBS and children with RAP.

## 2. Methods

**Figure 1:**
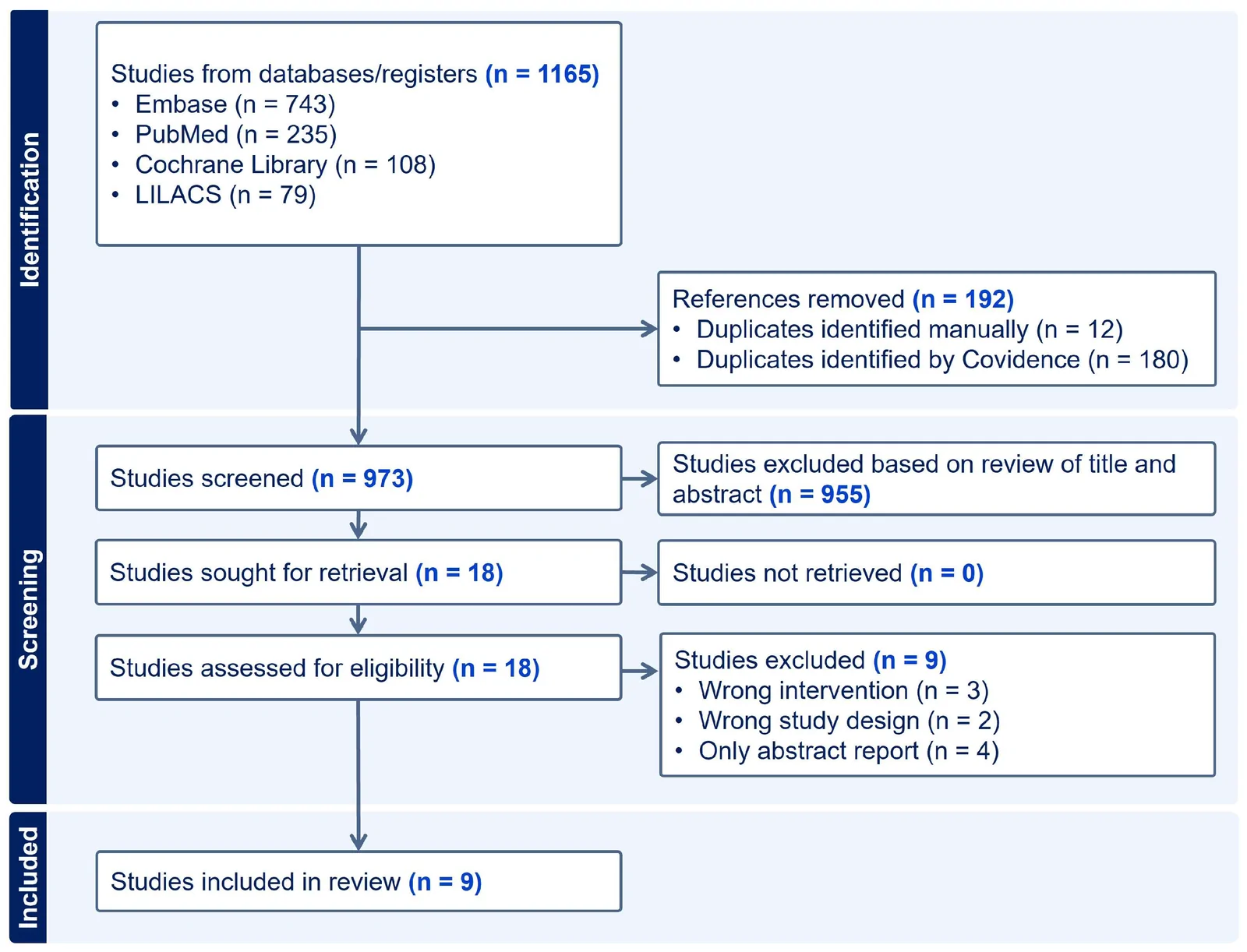
PRISMA chart representing the flow diagram of studies evaluated for this systematic review.

We conducted a systematic review of the literature following the Preferred Reporting Items for Systematic Reviews and Meta-Analyses (PRISMA) guidelines [23,24]. Figure 1 provides the flow diagram. The review protocol was registered in the International Prospective Register of Systematic Reviews (PROSPERO) (CRD42024617161). The PRISMA checklist is available as Supplementary Files.

### 2.1. Search Strategy and Study Selection

The search strategy was performed by LV according to a pre-specified protocol and included systematic inquiries in Embase, Pubmed, LILACS and Cochrane Library, with the help of a librarian with expertise in systematic reviews. Keywords consisted of ‘irritable bowel syndrome (MeSH)’, ‘IBS (tiab)’, ‘spastic colon (tiab)’ or ‘abdominal pain (Mesh:NoExp)’, ‘recurrent abdominal pain (tiab)’, ‘functional abdominal pain (tiab)’, in combination with any of the following: ‘lactose (MeSH)’, ‘dairy products (MeSH)’, ‘lactose reduc* (tiab)’, ‘lactose-free (tiab)’, ‘lactose avoidance (tiab)’, ‘milk avoidance (tiab)’, ‘low lactose (tiab)’ or ‘dietary interventions (tiab’). The detailed search strategy is provided in Supplementary File S1. The search was last updated on 15 August 2026 for potential articles published since the initial search.

Inclusion criteria were randomised controlled trials, prospective or retrospective cohort studies, and crossover and cross-sectional studies, with a minimum of 4 weeks of intervention or follow up. No restriction on publication time, language or geographical location was made. Exclusion criteria were case–control studies, case series/case reports, and studies with a lactose restriction for less than 4 weeks or a washout period of less than 4 weeks in a crossover trial.

Results were merged using Covidence software 2025 (Covidence systematic review software, Veritas Health Innovation, Melbourne, Australia). Following removal of duplicates, 3 reviewers (LV, MV and CM) examined the abstracts. The full-text articles of retained studies were analysed by 3 reviewers (LV, CM and SK) for eligibility. Each investigator reviewed the abstracts or full-text articles independently. Disagreements were resolved by consensus or consulting with a senior investigator.

### 2.2. Data Extraction

Two reviewers (LV and MV) performed the data extraction following the pre-specified protocol (Supplementary File S2). This included data on study design, patient characteristics, criteria for diagnosis of IBS and RAP, LM testing, and outcome-specific data such as symptoms, changes in quality of life, tolerance to the dietary intervention and absenteeism.

### 2.3. Outcome Variables

The primary outcome measure was a change compared to baseline in abdominal pain or discomfort after a dietary intervention aimed at reducing the dietary intake of lactose.

The secondary outcome measures were changes compared to baseline in stool frequency or consistency, bloating, abdominal distension, validated IBS severity questionnaires, and quality of life. Additional measures consisted of number of visits to healthcare professionals, tolerance to the dietary intervention and days of work lost.

### 2.4. Statistical Analysis and Presentation of Results

Risk of bias was assessed independently by 2 reviewers (MR and HD). For randomised controlled trials, the risk of bias was investigated with the Risk of Bias 2.0 tool (RoB2.0) [25]. For non-randomised studies, the risk of bias was ascertained by the Risk of Bias in Non-randomised Studies of Interventions (ROBINS-I v2) tool.

No meta-analysis was performed, because only one study provided a quantitative measure of pain frequency and because the control group differed from the study group in all but one study. All other studies only provided the frequency of patients with symptomatic improvement vs. baseline. Because effect sizes or variances were not reported, assessment of heterogeneity was not achievable.

## 3. Results

### 3.1. Description of the Study Cohorts

The literature search yielded 1165 records (Figure 1). After removal of duplicates, 973 records were reviewed based on title and abstract, of which 18 full-text articles were reviewed. Four studies were only available as an abstract. In three studies, the intervention did not consist of a lactose-free diet, and the study design did not correspond to the inclusion criteria in two studies. No randomised controlled trial fulfilled the inclusion criteria. Therefore, based on eligibility criteria, nine studies were included in the systematic review. Three prospective cohort studies investigated the effect of lactose-free diet (LFD) in IBS [26,27,28] in adult patients, and five prospective studies and one retrospective study investigated the effect on RAP in children [16,29,30,31,32,33]. All three studies on IBS were performed in Europe, whilst the six paediatric studies on RAP were performed in different parts of the world. Three studies were conducted in Europe [30,32,34], two in the USA [30,32] and one in Asia [16]. An overview of study characteristics is provided in Table 1.

One study in patients with IBS was published in the previous century. The older age of the studies is reflected by the outdated definitions of IBS based on the Manning and Rome II criteria. In these studies, a total of 224 subjects were included in the study group and 84 in a control group. Baños et al. studied the effects of an LFD for 6 months solely in patients with demonstrated LM based on a 25 g lactose H_2_BT [26]. Böhmer et al. and Vernia et al. compared the response to LFD for 6 weeks and 3 months, respectively, in patients with and without malabsorption based on 50 g and 25 g lactose H_2_BT, respectively [27,28]. Böhmer et al. additionally reported on the evolution in 22 IBS patients with positive H_2_BT not following a dietary intervention [27].

All but two of the paediatric studies on RAP were published before 2000 [29,30,32,33]. Only Ockeloen et al. diagnosed RAP according to the Rome III criteria [31]. All other studies based the diagnosis upon the presence of RAP with no apparent explanation from clinical examination, biochemistry, stool testing or radiological examination when indicated. Altogether, 231 subjects were included in the study group compared to 221 in the control group. Two studies compared the effects of an LFD in RAP patients with vs. without malabsorption [29,32]. One study compared LFD in patients with vs. without RAP [30]. Three studies did not include a control group [16,31,33]. The duration of LFD varied from 4 weeks up to 2 years.

### 3.2. Prevalence of Lactose Intolerance or Malabsorption in Patients with IBS or RAP

The presence of LM in patients was 24% in two studies in IBS [26,27], despite using a different dose of lactose for the breath test. Malabsorption was remarkably higher in IBS patients in the study of Vernia (68.2%), but intolerance was reported in 22.6% of participants [28]. In children with RAP, the prevalence of LM was highly variable (24–75%), despite similar lactose dosages for the lactose breath test (2 mg/kg) in all but one study (1 mg/kg).

In contrast, LM was present in 5.7–20% in healthy volunteers in adults [26,27] and 25% in children with abdominal symptoms not fulfilling the criteria for RAP [30].

### 3.3. Primary Outcome: Change in Abdominal Pain or Discomfort

An overview of the reduction in abdominal pain or discomfort in response to an LFD in adults with IBS and children with RAP is given in Table 2. Overall, outcome measures were poorly defined. Three studies reported on improvement after an LFD without further details [16,26,33]. Studies reported on an unspecified improvement in pain [30], an improvement based on the judgment from the physician [29], or as “not meeting the definition of functional abdominal pain” [31]. Two studies reported on the frequency of patients with improvement in abdominal pain [28,32]. Only Ceriani et al. provided specific data on the decrease in the frequency—not severity—of abdominal pain [29].

Notwithstanding the highly disparate outcome measures, patients with IBS or RAP appear to have experienced improvement or resolution of pain during an LFD more frequently when LM was confirmed by LBT. Pain improvement or resolution was reported in 100% of IBS patients according to Vernia et al. [28], while pain scores decreased in the study from Böhmer [27]. In RAP, pain improved in 59–100% of subjects at 4 weeks to 3 months based on the results from five studies [16,29,30,31,32]. In contrast, pain improved by LFD in 0–32% of patients without demonstrated LM, as reported in three studies [29,30,32].

### 3.4. Secondary Outcomes

An overview of change in secondary outcomes in response to an LFD in adults with IBS and children with RAP is given in Table 2. “Complete symptom resolution” was achieved in 1 of 50 patients with IBS according to Baños et al. [26], but symptoms were not specified. No further details on the symptoms that were assessed were available from the manuscript. In patients with LM, Vernia et al. found a complete and partial remission of symptoms in 44% and 39%, respectively, of patients following an LFD compared to 18% of patients reporting partial remission without dietary intervention [28]. Similarly, in the same study, 25% of IBS patients without LM reported partial remission with an LFD. They also identified a decrease in bloating and diarrhoea in 43% and 58%, respectively, of patients reporting these symptoms during LBT. In the study from Böhmer et al., the cumulative symptom score decreased significantly, with 7.3 points at 3 weeks and 4.2 points at 6 weeks vs. baseline in patients with a positive LBT, but not in the control group, while the pain and abdominal distension score decreased only in patients with LM (58% and 44%) [27]. Webster et al. reported on a trend in improved diarrhoea and constipation in LM+ IBS patients vs. controls.

Long-term persistence of symptomatic improvement was assessed by two studies [31,33]. Improvement in symptoms persisted for 6 months in 88% of patients in the study from Winter et al. According to the retrospective study of Ockeloen et al., the effect on pain tended to decrease during prolonged follow-up (from 94% at 5 months to 61% at 15 months) [31].

### 3.5. Quality of Life, Healthcare Visits, Days of Work Lost, Tolerance to the Diet

None of the studies addressed quality of life, healthcare visits, days of work lost (either absenteeism or presenteeism), or tolerance or the adherence to the diet.

### 3.6. Risk of Bias

Overall, all studies demonstrated a critical risk of bias in several categories, as illustrated in Table 3. The risk of missing data was deemed critical for all but one study. All studies suffered from a high risk in bias due to confounding, measurement of outcomes and selection of reported results. Results were only reported on a subset of the population. Three harboured a serious risk of bias in the classification of the interventions, and three studies suffered from a serious bias in the selection of participants.

## 4. Discussion

According to the results of this systematic review, in the presence of malabsorption, as defined based on the result of the LBT, an LFD resulted in an improvement in pain in the majority of patients. However, results are tainted by the lack of high-quality evidence for the role of LFDs in patients suffering from IBS and RAP. In the nine eligible studies identified by the systematic search, diagnosis of IBS and RAP relied on old criteria, frequently predating the actual Rome definition [1,11]. LM was identified in 24–75% of patients with IBS or RAP. Distension/bloating and stool consistency improved according to three studies. However, the design of the studies and the outcome assessment of the symptoms bear several flaws, preventing firm conclusions. The absence of a true control group, blinded randomisation, well-defined outcome measures and the lack of assessment of patient adherence to the dietary intervention result in a high risk of bias.

Because of the lack of biomarkers, the diagnosis of IBS and RAP rely on symptom-based criteria. The Rome criteria require a minimal duration of abdominal pain and an association with altered bowel habits. Manning criteria lack the time criterion while including other symptoms such as passage of mucus and sensation of incomplete evacuation, which are absent in the Rome definitions. Later iterations of the Rome criteria required a minimal frequency of abdominal pain, while Rome IV abandoned abdominal discomfort in favour of abdominal pain. Earlier studies in RAP define the diagnosis based on more extensive testing than usually required by Rome criteria. Rome IV moved away from RAP as a diagnosis in children, and instead classifies children with chronic/recurrent abdominal pain into different functional abdominal pain disorders in analogy to the classification in adults. Therefore, it seems unlikely that results would differ if more recent criteria had been applied in these studies. Many of the children with the diagnosis of RAP would now be considered to have IBS according to the Rome V criteria [2].

The rationale for the refinement of the definitions stems from evolving understanding of the disease pathophysiology and relation to therapeutic options. Different studies evidenced the varying prevalence of IBS depending on these definitions [34,35,36,37]. Guidelines advocate different therapeutic options based on different subtypes [38,39,40]. Therefore, caution should be exercised when translating results from studies using older definitions of IBS into current clinical practice. Confirmation of earlier findings using recent criteria is recommended.

While pain represents the cardinal symptom of IBS, patients frequently experience other bothersome symptoms such as bloating, diarrhoea, and constipation. Management of IBS requires adequately addressing all these symptoms to ensure best outcomes and improvement of quality of life. For therapeutic interventions, both the US Food and Drug Administration (FDA) and the European Medical Agency (EMA) require binary endpoints [41], combining improvement in pain and bowel habits. None of the studies considered in this review fulfilled these criteria. In many cases, improvement or resolution remained undefined. When specific symptoms were addressed, pain was most assessed, while abdominal distension/bloating and diarrhoea were addressed by three studies. However, improvement in stool by LFD pattern remained inadequately addressed in the studies under consideration when compared to current recommendations.

In contrast to the disparities in outcome measures, the amount of lactose administered during H2BT was largely comparable to existing international recommendations of 25 g in adults and 2 g/kg in children [6,7]. In contrast, Böhmer et al. used a dose of 50 g [27], while only 1 g/kg was given by Webster et al. [32].

Our systematic review also revealed other qualitative issues of these studies. Half of them did not include a control group. When included, subjects and investigators were not blinded to the results of the H2BT. Addressing the role of an LFD in relation to the presence of malabsorption requires a different approach. Symptomatic improvement should be compared between malabsorbers and non-malabsorbers, as well as intolerant and non-intolerant subjects, with groups remaining blinded to the results of the lactose H2BT. Ideally, the study protocol should include an evaluation of adherence to the diet. Indeed, dietary changes may result in symptom improvement irrespective of test results. Using this approach, Melchior et al. demonstrated the effectivity of a low-fructose diet independently of the presence of fructose malabsorption, as evidenced by breath testing [42].

Patient expectation plays a major role in symptom generation. Seiler et al. showed that IBS patients with self-perceived gluten sensitivity reacted similarly to gluten, wheat, and sham challenges [43]. Concerning lactose, in their systematic review, Pop et al. revealed a high proportion of negative lactose H2BT in patients with self-reported LI [22]. In the studies included in this review, knowledge of the results of the lactose H2BT by investigators, patients or their parents might result in bias about the response to an LFD. It is therefore recommended that patient adherence be confirmed when studying the role of dietary interventions in IBS, ideally by providing the meals or by food diaries, while ensuring adequate measures for double-blind assessents.

Current IBS management guidelines recommend against systematic testing for carbohydrate malabsorption, such as LM testing, and favour a positive diagnosis [1,11,38,40]. Concerning the paediatric population, a Cochrane analysis published in 2009 concluded that there is insufficient evidence for a low-lactose diet in the management of children with RAP [44,45]. Nevertheless, healthcare professionals commonly order lactose breath testing when evaluating a patient with suspected IBS [46]. Different reasons potentially explain this. Patients frequently believe that food is the trigger and expect confirmation by a diagnostic mean. This is reflected by high rates of IBS patients with severe food avoidance and restrictions [47]. During the diagnostic process, patients and healthcare professionals rely on the exclusion of organic disorders, with a carbohydrate H2BT representing an accessible test. However, in accordance with Rome recommendations, a positive diagnosis should be favoured. Therefore, ordering an LBT should be restricted to patients where history strongly suggests a link between lactose intake and symptoms. Additionally, intolerance should rely on the presence of malabsorption in association with symptoms during the test [6,7].

### Strengths and Limitations

Different strengths of the current systematic review warrant highlighting. To minimise selection bias, different measures were taken. There were no restrictions on the country of origin or the language of publication. A librarian experienced in systematic search helped define the search strategy, which was deployed in four large databases. Including both IBS and RAP in one review process ensured inclusion of studies across all age categories. This resulted in over 1000 abstracts screened for eligibility. Additionally, data extraction was performed according to a predetermined protocol, which was registered on PROSPERO, to ensure qualitative and completeness of the review.

Nevertheless, some limitations should be taken into consideration. First, we did not contact the authors of the included studies because of the date of publication. The other limitations are mainly due to the nature of the included studies. Attrition bias is probable, as reflected by the fact that many patients were lost to follow-up in several studies, while the design of the studies as prospective cohorts suggests a possible reporting bias. Also, none of the reviewed studies reported a sample size calculation to determine the adequate population size. Finally, the heterogeneity in reported outcome measures between studies and the absence of quantitative data precluded a meta-analysis of the efficacy of LFD in IBS and RAP.

## 5. Conclusions

In conclusion, pain associated with IBS and RAP improves when committing to a lactose-free diet when LM is present. Similar to the general population, LM evidenced by lactose H2BT is reported in 24–75% with IBS or RAP, based on the results of studies involving prospective cohorts of patients. However, the studies included in this analysis are at a high risk of bias. Diagnosis relied on outdated criteria, while outcome measures remained limited to improvement in pain. The lack of a control group with a negative LBT and the absence of blinding for the results of LBT further limits the generalisability of the role of LFDs in IBS and RAP. Impact on bowel habits, which represents an important aspect in IBS, remained unexplored. Many studies lacked a valid control group.

Future studies are needed to validate the role of an LFD in the management of abdominal pain in IBS and RAP and its relationship to the result of lactose H2BT. These studies should compare the response of individual symptoms as well as a composite endpoint to an LFD in patients, while ensuring that patients and investigators remain blinded to the result of the LBT.

## Figures and Tables

**Table 1:** Study characteristics and results from studies of lactose malabsorption in adults with irritable bowel syndrome and children with recurrent abdominal pain.

Study	Year	Country	Definition	Design	Study Group	Control Group	Diagnosis of Malabsorption	Intervention, Duration (n)
Baños [26]	2005	Spain	IBS Rome II	Prospective cohort	50 LM+ IBS	--	25 g H_2_BT	LFD, 6 months
Böhmer [27]	2006	The Netherlands	IBS Manning	Prospective cohort	17 LM+ IBS	50 LM- IBS	50 g H_2_BT	LFD, 3 and 6 weeks
Vernia [28]	1995	Italy	IBS Manning	Prospective cohort	157 LM+ IBS	12 LM- IBS 22 LM+ no diet	25 g H_2_BT	LFD, 3 months
Ceriani [29]	1988	Italy	RAP Pre-Rome	Prospective cohort	18 LM+ RAP	4LM- RAP	2 g/kg H_2_BT	LFD, 3 months
Garg [16]	2017	India	RAP Pre-Rome	Prospective cohort	34 LM+ RAP	--	2 g/kg H_2_BT	LFD, 4 weeks
Liebman [30]	1979	USA	RAP Pre-Rome	Prospective cohort	38 RAP+	29 RAP-	2 g/kg H_2_BT	LFD, 4 weeks and 1 year
Ockeloen [31]	2012	The Netherlands	RAP Rome III	Retrospective cohort	91 RAP+	--	2 g/kg H_2_BT	LFD, 5 and 15 months
Webster [32]	1995	USA	RAP Pre-Rome	Prospective cohort	33 LM+ RAP	104 LM- RAP	1 g/kg H_2_BT	LFD, 3 months to 2 years
Winter [33]	1990	Germany	RAP Pre-Rome	Prospective cohort	17 LM+ RAP	--	2 g/kg H_2_BT	LFD, 6 months

H_2_BT: hydrogen breath testing; IBS: irritable bowel syndrome; LFD: lactose-free diet; LM+: lactose malabsorbers according to results of lactose hydrogen breath test; LM-: normal lactose absorption; RAP: recurrent abdominal pain.

**Table 2:** Primary outcome of lactose-free diet in irritable bowel syndrome and recurrent abdominal pain. ↓ decrease in the respective symptom score.

Author	% Malabsorption	Outcome Measures	Primary Outcome (Pain)	Secondary Outcomes
Baños [26]	24% in IBS 20% in health	Unspecified	N/A	1/50 complete resolution of symptoms
Böhmer [27]	24.3% in IBS 5.7% in health	Decrease in symptoms and cumulative score	Decrease in pain score in LM+ IBS	↓ in cumulative symptom score, distension, diarrhoea, in LM+ IBS
Vernia [28]	68.2% in IBS	Improvement in symptoms of pain, distension, diarrhoea, mucus, general condition	10/19 complete “remission of symptoms” and in 9/19 partial “remission of symptoms” in 19 patients experiencing abdominal pain during LBT No data on effect on pain for the total group	↓ in bloating in 18/42 (43%) when symptoms during LBT; ↓ in diarrhoea in 7/12 (58%) when symptoms during LBT. LM+ and diet; 48/110 (44%) complete “remission of symptoms”, 43/110 (39%) partial “remission of symptoms” LM- and diet: 3/12 (25%) partial “remission of symptoms” LM+ and no diet: 4/22 (18%) partial “remission of symptoms”
Ceriani [29]	75% in RAP	Improvement assessed by physician	Mean number of pain episodes per month in LM+ after LFD: 16.9 ± 11.5 vs. 1.6 ± 1.2 14/18 (78%) LM+ improvement in pain 4/18 LM+ (22%) with 40% decrease in pain 1/4 LM- (25%) subjective improvement in pain	
Garg [16]	58% in RAP	Unspecified improvement	20/34 (59%) LM+ pain improvement	
Liebman [30]	29% in RAP 25% in RAP-	Unspecified total pain relief	Pain improvement in 10/11 (91%) LM+ at 6 weeks, 9/11 (82%) at 1 year, and 0/6 LM- No change in pain in 1/1 LM+ in control group	
Ockeloen [31]	59% in RAP	Not meeting definition of functional abdominal pain	5 months: pain improvement In 17/18 (94%) LM+ 15 months: pain improvement in 11/18 (61%) LM+	
Webster [32]	24% in RAP	Symptom score (pain, bloating, gas, flatulence, diarrhoea, constipation)	LM+:20/27 (74%) resolution, less frequent abdominal pain LM-:28/88 (32%) resolution of pain	Trend in improvement in diarrhoea and constipation in LM+ vs. controls
Winter [33]	28.3% in RAP	Unspecified improvement	N/A	1 month: 16/17 (94%) improved 6 months: 15/17 (88%) improved

IBS: irritable bowel syndrome; LFD: lactose-free diet; LM+: lactose malabsorbers according to results of lactose hydrogen breath test; LM-: normal lactose absorption; N/A: not assessed; RAP: recurrent abdominal pain; RAP-: child without RAP, but with gastrointestinal problems.

**Table 3:** Assessment of the risk of bias according to ROBINS-2.0.

Study	D1	D2	D3	D4	D5	D6	Overall						
Baños [26]										Critical			
Böhmer [27]										Serious			
Ceriani [29]										Moderate			
Garg [16]										Low			
Liebman [30]													
Ockeloen [31]									D1	Bias due to confounding			
Vernia [28]									D2	Bias in classification of interventions			
Webster [32]									D3	Bias due to selection of participants			
Winter [33]									D4	Bias due to missing data			
									D5	Bias in measurement of outcomes			
									D6	Bias in selection of the reported results			

## Data Availability

Dataset available on request from the authors.

## Supplementary Materials

The following supporting information can be downloaded at: https://www.mdpi.com/article/10.3390/nu18213619/s1. File S1: Detailed search strategies, File S2: Overview of the data extracted from eligible articles according to the protocol. File S3: Prisma checklist, File S4: Prisma abstract checklist.

## Abbreviations

EMA	European Medical Agency
FDA	US Food and Drug Administration
H2BT	hydrogen breath test
IBS	irritable bowel syndrome
LBT	lactose breath test
LFD	lactose-free diet
LI	lactose intolerance
LM	lactose malabsorption
PROSPERO	Prospective Register of Systematic Reviews
RAP	recurrent abdominal pain
