# Supplementary material for: Efficacy of Lactose Reduction in Irritable Bowel Syndrome and Recurrent Abdominal Pain: A Systematic Review
Source: Nutrients. 2026 Oct 31;18(21):3619. doi: 10.3390/nu18213619 (PMC13764785; doi:10.3390/nu18213619)
Supplement: Supplementary file 1 [file nutrients-18-03619-s001.zip › supplementary file S1 and S2.pdf]

Supplementary File S1. Detailed search strategies

Lilacs search strategy

((ibs) OR (irritable bowel syndrome) OR (spastic colon) OR (recurrent abdominal pain) OR (abdominal pain) OR (functional abdominal pain)) AND ((lactose) OR (dairy products) OR (lactose reduc*) OR (lactose free) OR (lactose avoidance) OR (milk avoidance) OR (dairy avoidance) OR (low lactose) OR (dietary interventions))

Embase search strategy

#21 #19 AND #20

#20 #9 OR #10 OR #11 OR #12 OR #13 OR #14 OR #15 OR #16 OR #17 OR #18

#19 #1 OR #2 OR #3 OR #4 OR #5 OR #6 OR #7 OR #8

#18 '**dietary interventions**':ti,ab

#17 '**low lactose**':ti,ab

#16 '**dairy avoidance**':ti,ab

#15 '**milk avoidance**':ti,ab

#14 '**lactose avoidance**':ti,ab

#13 '**lactose free**':ti,ab

#12 '**lactose reduc***':ti,ab

#11 '**dairy product**'/mj

#10 '**lactose**'/exp

#9 '**lactose**'/mj

#8 '**functional abdominal pain**':ab,ti

#7 '**abdominal pain**'/mj

#6 '**recurrent abdominal pain**':ab,ti

#5 '**recurrent abdominal pain**'/exp

#4 '**spastic colon'**:ab,ti

#3 '**irritable colon**':ab,ti

#2 '**irritable colon**'/exp

#1 **ibs**:ab,ti

Cochrane search strategy

#1 MeSH descriptor: [Irritable Bowel Syndrome] explode all trees

#2 (IBS):ti,ab;kw

#3 (spastic colon):ti,ab,kw

#4 MeSH descriptor: [Abdominal Pain] explode all trees

#5 ("recurrent abdominal pain"):ti,ab,kw

#6 ("functional abdominal pain"):ti,ab,kw

#7 MeSH descriptor: [Lactose] explode all trees

#8 MeSH descriptor: [Dairy Products] explode all trees

#9 (lactose reduc*):ti,ab,kw

#10 (lactose free):ti,ab,kw

#11 (lactose avoidance):ti,ab,kw

#12 (milk avoidance):ti,ab,kw

#13 (dairy avoidance):ti,ab,kw

#14 (low lactose):ti,ab,kw

#15 #1 OR #2 OR #3 OR #4 OR #5 OR #6

#16 #7 OR #8 OR #9 OR #10 OR #11 OR #12 OR #13 OR #14

#17 #15 AND #16

<u>PubMed search strategy</u>

#17 #15 AND #16

#16 #7 OR #8 OR #9 OR #10 OR #11 OR #12 OR #13 OR #14

#15 #1 OR #2 OR #3 OR #4 OR #5 OR #6

#14 "low lactose"[tiab]

#13 “dairy avoidance"[tiab]

#12 "milk avoidance"[tiab]

#11 "lactose avoidance"[tiab]

#10 "lactose free"[tiab]

#9 "lactose reduc*"[tiab]

#8 "dairy products"[Mesh]

#7 "Lactose"[Mesh]

#6 "functional abdominal pain"[tiab]

#5 “Abdominal Pain"[Mesh:NoExp]

#4 “recurrent abdominal pain"[tiab]

#3 "spastic colon"[tiab]

#2 "IBS"[tiab]

#1 “irritable bowel syndrome"[MeSH Terms]

Supplementary file S2: Overview of the data extracted from eligible articles according to the protocol:

Patient specific	Age<br>Gender<br>Ethnicity<br>Lactose intake at baseline and during dietary intervention<br>Genetic testing for lactase deficiency
Diagnostic criteria	For Irritable Bowel Syndrome<br>For Recurrent Abdominal Pain
Diagnosis of lactose malabsorption	Test modality<br>Diagnostic criteria<br>Concurrent symptom registration during the test
Outcome specific	Abdominal pain<br>Abdominal discomfort<br>Bloating<br>Abdominal distension<br>Stool consistency<br>Stool frequency<br>Number of visits to healthcare professionals<br>Work loss<br>Tolerance to dietary intervention
Study specific	Study design<br>Sample size<br>Country<br>Duration of lactose restriction<br>Assessment of adherence to dietary intervention

Supplementary File S3: PRISMA checklist

See separate word document
